# Gamma Radiation-Induced Advanced 2,3-Dimethylacrylic Acid-(2-Acrylamido-2-methyl-1-propanesulfonic Acid) Superabsorbent Hydrogel: Synthesis and Characterization

**DOI:** 10.3390/gels9050426

**Published:** 2023-05-19

**Authors:** Md Murshed Bhuyan, Jae-Ho Jeong

**Affiliations:** Thermal-Fluid Energy Machine Laboratory, Department of Mechanical Engineering, Gachon University, 1342 Seongnam-daero, Sujeong-gu, Seongnam-si 13120, Gyeonggi-do, Republic of Korea

**Keywords:** hydrogel, gamma radiation, swelling, DMAA, AMPSA

## Abstract

Gamma radiation technique for the preparation of pure hydrogels is gaining popularity worldwide. Superabsorbent hydrogels play vital roles in different fields of application. The present work mainly focuses on the preparation and characterization of 2,3-Dimethylacrylic acid-(2-Acrylamido-2-methyl-1-propane sulfonic acid) (DMAA–AMPSA) superabsorbent hydrogel by applying gamma radiation and optimization of the proper dose. To prepare DMAA–AMPSA hydrogel, different doses ranging from 2 kGy to 30 kGy were imparted on the blend aqueous solution of the monomers. The equilibrium swelling increases with increasing radiation dose, followed by decreasing after reaching a certain level, and the highest result is found to be 26,324.9% at 10 kGy. Fourier Transform Infrared (FTIR) and Nuclear Magnetic Resonance (NMR) spectroscopy confirmed the formation of co-polymer by showing the characteristic functional groups and proton environment of the gel. X-ray Diffraction (XRD) pattern indicates the crystalline/amorphous nature of the gel. The Differential Scanning Calorimetry (DSC) and Thermogravimetry Analysis (TGA) revealed the thermal stability of the gel. The surface morphology and constitutional elements were analyzed and confirmed by Scanning Electron Microscopy (SEM) equipped with Energy Dispersive Spectroscopy (EDS). Finally, it can be stated that hydrogels can be usable in metal adsorption, drug delivery, and other relevant fields.

## 1. Introduction

Hydrogels are polymeric materials capable of retaining a large amount of water (solvent) in their structure. The hydrogels, which can hold water 100 times their dried weight, are referred to as superabsorbent hydrogels (SH). The necessities of SHs are in improving efficiency in applications [1,2]. They exhibit extra-ordinary hydrophilicity due to the presence of carboxylic acid (–COOH), hydroxyl (–OH), amide (–CONH_2_), nitro (–NO_2_), sulfonic acid (–SO_3_H), and phosphonic acid (–PO_3_H) functional groups [3]. The half-liquid and half-solid properties facilitate the relaxation behavior of hydrogel resulting in the stimuli-responsive-like controlled swelling in an ionic medium, electric field, pH, and temperature [4]. Due to the extensive area of application, hydrogels are getting concern worldwide. A few of the SHs are very effective in selective metal adsorption [5], drug delivery [6], dye adsorption [7], wound dressing [8], and biomedical applications [9]. The essential properties of hydrogels are swelling, mechanical strength, and re-usability, which can be enriched through grafting/cross-linking between different monomers/polymers [10,11]. SHs encounter irreversible damage after de-swelling, which can be attributed to the absence of a suitable energy dissipation mechanism. This limitation can be overcome by increasing the H-bonding interactions and the covalent bond in the hydrogel networks [12]. A 2-acrylamido-2-methyl-1-propanesulfonic acid (AMPSA) is a solid electrolytic monomer with both the amide and sulfonic acid functional groups that can easily form H-bonds. AMPSA is used to prepare pH-independent superabsorbent hydrogel with various monomers/polymers and homo-polymer-poly(AMPSA) [13]. Poly (AMPSA) is a kind of superabsorbent hydrogel having applications in different fields but not mechanically strong enough to show reversible swelling behavior [14]. A 2,3-Dimethylacrylic acid (DMAA, also known as Tiglic acid) is a monomer having a monocarboxylic acid functional group and is found to be a natural product. DMAA possesses a double bond between carbons 2 and 3 of its structural formula, which can be broken to form a homopolymer/copolymer. Until now, it has not been used extensively in hydrogel preparation. DMAA can be incorporated with AMPSA to prepare a mechanically strong hydrogel by increasing the number of H-bonding. Multiple attempts were made to improve the reversible swelling and the mechanical strengths of the hydrogels. The preparation method plays an important role in the purity and functionality of the hydrogels from their respective monomers/polymeric raw materials [15]. There are several methods of hydrogel preparation, including bulk, solution/cross-linking, suspension/grafting, and radiation polymerization [16]. The radiation techniques use UV-visible [17], microwave [18], and gamma rays [19] for the initiation of the reaction. Esra Su et al. prepared self-healing poly (AMPSA) by applying UV-radiation without using a cross-linker which gave a maximum swelling capacity of 1700 g.g^−1^ [12]. Shwetha Krishna Murthy et al. reported microwave-assisted Chitosan/Polycarbophil super-porous hydrogels, which do not show better swelling, and the polymerization requires cross-linking agent Vanillin [20]. Despite the conventional methods, the UV-visible (200–700 nm wavelength) and the microwave (1 mm–30 cm wavelength) types of radiation are not highly energetic to activate all of the monomers transferring into the transition state where the reaction starts to proceed. Subsequently, the gel content and swelling of the hydrogel are not as much as expected. Gamma radiation is highly energetic (<0.25 Å wavelength, >12 EHz (1 EHz = 10^18^ Hz) and >50 keV energy) ionizing ray expressed in Gray unit (1 Gy (100 rad) = 1 J/kg). The sources of gamma radiation are Potassium-40 and Co-60, whereas the latter one is synthetic and can be produced by bombarding Co-59 with a slow neutron, as shown in the nuclear reaction [21].

_27_Co^59^ + n → _27_Co^60^ → _28_Ni^60^ + e^−^ + ν^−^_e_ + gamma rays (1)

Gamma radiation is usually used in refining [22], mining [23], textile [24], pulp, and paper industries [24]. The most significant feature of gamma-radiation-induced polymerization is not to use of initiator cross-linking agents resulting in the formation of pure products. Gamma radiation initiates the reaction within a short span of time and yields maximum gel contents compared to the other electromagnetic radiations resulting in the best choice for hydrogel synthesis. Fengyi Chen et al. reported on the gamma-radiation-induced starch–acrylamide hydrogel for releasing urea in the agricultural field, but the swelling was not studied significantly [25]. Using a cross-linking agent and initiator, AMPSA/Acrylic acid and AMPSA/acrylamide hydrogels were reported where the hydrogels are neither pure nor showing better water absorbency [26,27]. Therefore, the combination of monomers is deserving, which can undergo co-polymerization easily upon irradiation, be able to retain a large amount of water, and fits the other characteristics well. The monomers DMAA and AMPSA may suit the requirements. However, the preparation of co-polymeric hydrogels from DMAA and AMPSA by applying gamma radiation has not yet been studied. The objective of the present work is to synthesize DMAA–AMPSA hydrogel by applying different doses of gamma radiation, characterization, and optimizing radiation dose.

## 2. Results and Discussion

### 2.1. Radiation Polymerization of DMAA-AMPSA

**Scheme 1:**
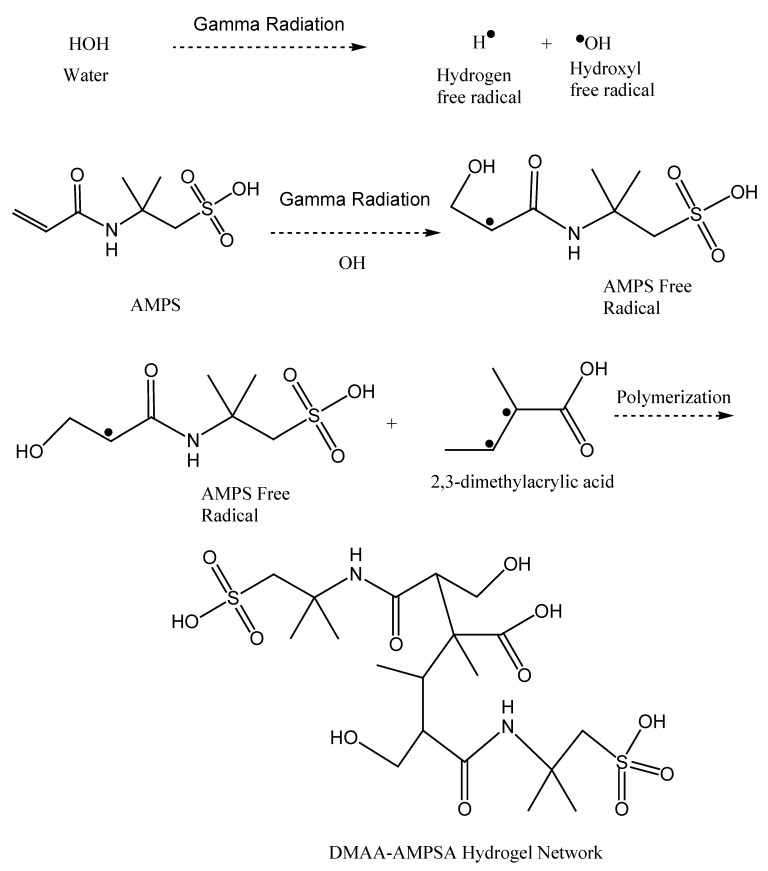
Probable polymerization of DMAA–AMPSA hydrogel.

Gamma radiation is a highly energetic electromagnetic radiation capable of producing free radicals from vinylic organic monomers [28]. The gamma-radiation-induced polymerization was explained in detail by J. M. Rosiak and P. Ulanski [29]. Esmaiel Jabbari et al. also described the y-radiation cross-linking of polyacrylic acid in an aqueous solution [30]. Here, the probable reaction mechanism is explained shortly. Copolymerization between DMAA and AMPSA was performed by free radical polymerization in an aqueous blend solution containing no initiator and the cross-linking agent. Both the DMAA and AMPSA possess a vinylic part in their structure which can be turned into free radicals upon irradiating gamma rays. First, the water molecule undergoes hydrogen and hydroxyl free radical formation upon gamma-ray irradiation, where the two hydrogen-free radicals combine to produce hydrogen gas and escape. Then, the hydroxyl free radicals combine with AMPSA to produce their own free radicals at the adjacent pie bond point [31]. The free radicals propagate and terminate to produce the final product hydrogels. Scheme 1 represents the probable free radical mechanism of hydrogel formation. Since there is no external cross-linking agent added, the smaller monomer DMAA may act as a self-cross linker between two copolymer/homopolymers of DMAA and AMPSA, followed by the formation of the final product.

### 2.2. Effect of Radiation Dose on Equilibrium Swelling of Gels

**Figure 1:**
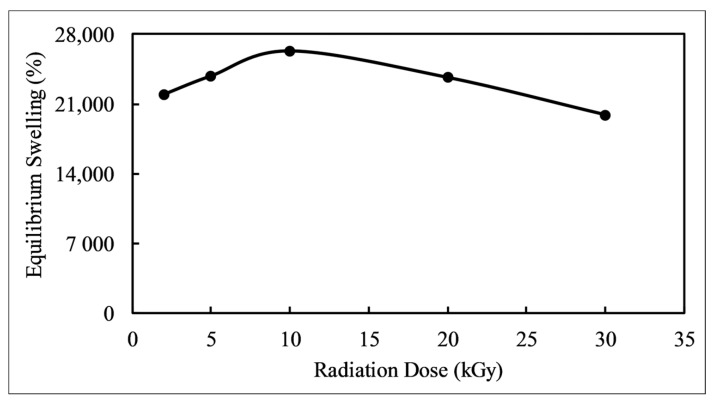
Effect of gamma radiation dose on equilibrium swelling of hydrogel.

**Figure 2:**
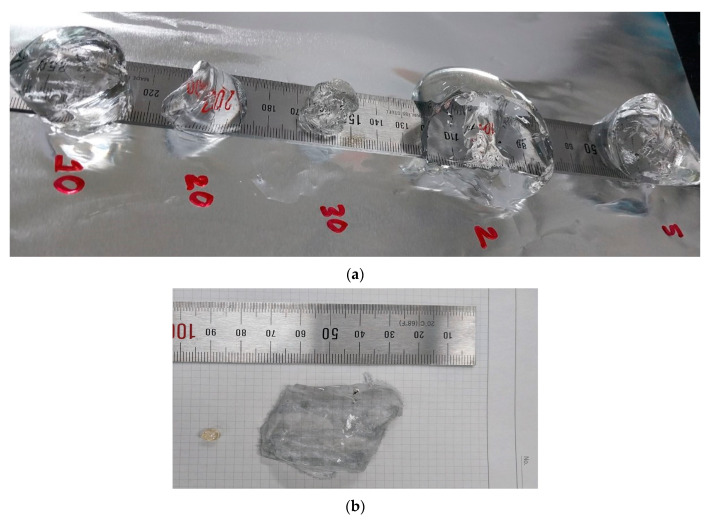
DMAA–AMPSA hydrogels (**a**) swelled hydrogels prepared at 2, 5, 10, 20, and 30 kGy. doses and (**b**) prepared at 10 kGy dose (before and after swelling).

Equilibrium swelling predicts the hydrogel’s performance and application in different fields of study. Figure 1 depicts the effect of gamma radiation dose on the equilibrium swelling of hydrogels, where the swelling increases with increasing radiation dose up to 10 kGy, followed by a decreasing trend up to 30 kGy. This change can be attributed to the lower dose being unable to activate all of the monomers to move into a transition state for the reaction to begin [32]. At 10 kGy radiation dose, the maximum amount of monomers undergo forming free radicals as well as getting activated to form the copolymer. Subsequently, the highest number of functional groups are present in the hydrogel, which is responsible for the optimum swelling. Above the optimum radiation dose (10 kGy), the monomers undergo degradation, which results in a declining degree of copolymerization and equilibrium swelling. Thus, the equilibrium swelling is lower at a 30 kGy dose. Figure 2 exhibits the dried and swelled states of the hydrogels at neutral pH and room temperature. Figure 2a shows the swollen state hydrogels prepared by applying 2, 5, 10, 20, and 30 kGy radiation doses, where it is obvious that the hydrogel prepared at 10 kGy radiation dose undergoes the maximum swelling compared to the other samples. The equilibrium swelling gives better results compared to other hydrogels because of the presence of several functional groups such as –COOH, –SO_3_H, –NH_2_, etc. Moreover, the void spaces among the polymeric network may be higher and more sequential than in hydrogels of other radiation doses. At neutral pH, these groups can easily form a greater number of hydrogen bonds with water molecules which results in retaining large amounts of water and greater swelling. Table 1 lists the recently reported equilibrium swelling of a few AMPSA-based hydrogels.

Figure 2b reflects the scale bar of the dried and swollen state of hydrogel prepared at a 10 kGy dose evaluating the maximum equilibrium swelling of 26,324.9% of dried gel, which is much better than previously prepared hydrogels. Therefore, on the basis of swelling, the hydrogels can be categorized as superabsorbents and can be used in different fields of application.

### 2.3. Characterization of Hydrogel by FTIR Spectroscopy

**Figure 3:**
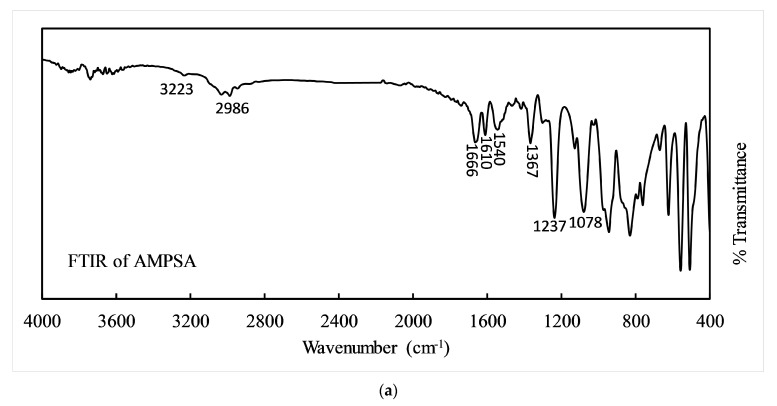

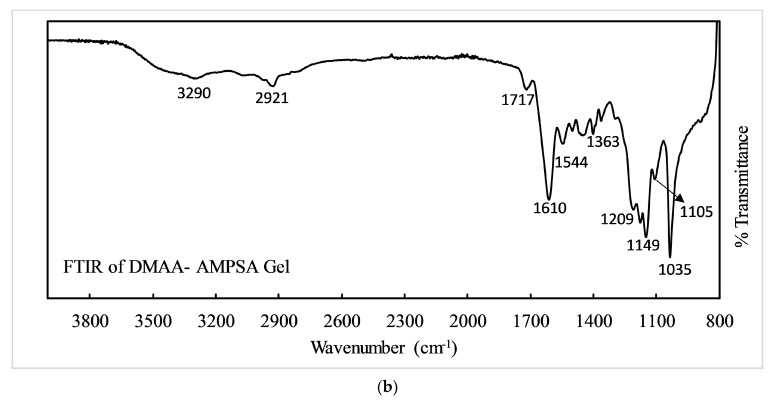
FTIR spectrum of (**a**) AMPSA powder and (**b**) DMAA-AMPSA gel.

To clarify the existing functional groups in the polymeric gel and to confirm the polymerization, FTIR spectra were carried out with KBr calibration and presented in Figure 3. Davide Ferri et al. reported on the characteristics of FTIR peaks of DMAA [39]. Here, the FTIR spectra of AMPSA and gel are presented and analyzed to confirm the copolymerization. Figure 3a shows the FTIR of pristine AMPSA powder, where peaks at 3223 cm^−1^ and 2986 cm^−1^ are for –N–H and –C–H stretching vibrations. The characteristic peaks at 1666 to 1610 cm^−1^, 1540 cm^−1^, 1367 cm^−1^, 1237 cm^−1^, and 1078 cm^−1^ are attributed to carbonyl (–C=O) stretching of –CONH-, –N–H bending, –C–N stretching, and symmetric and asymmetric peaks of the sulfonic acid group explained in gels spectrum [40]. Due to copolymerization, the strength of peaks may change to some extent. In Figure 3b, the peaks at 3290 cm^−1^ and 2921 cm^−1^ are for the –O–H stretching overlap with the –N–H peak and –C-H stretching of the carbohydrate chain. The peaks at 1717 cm^−1^ and 1610 cm^−1^ are attributed to –C=O stretching of carboxylic acid (–COOH) and acyl amino (–CONH-) groups, indicating the presence of DMAA and AMPSA. The characteristic peaks at 1363 cm^−1^, 1209 cm^−1^, 1149 cm^−1^ (strong band), 1105 cm^−1^, and 1035 cm^−1^ (strong band) correspond to –C–N stretching, asymmetric stretching of -SO_2_, symmetric stretching of –SO_2_, –SOH stretching, and –S–O stretching of –SO_3_H [26]. Therefore, FTIR confirmed the polymeric hydrogel formation between DMAA and AMPSA.

### 2.4. Nuclear Magnetic Resonance (NMR) Spectroscopy

**Figure 4:**
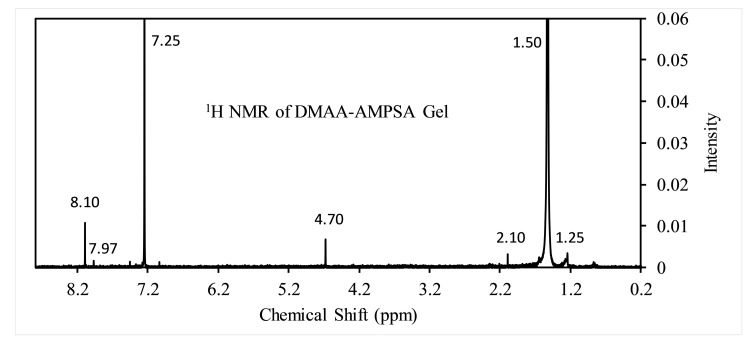
^1^H NMR of DMAA–AMPSA hydrogel.

Proton nuclear magnetic resonance (^1^H NMR) spectrum of gel carried out in chloroform solvent to observe the different environments of the proton. ^1^H NMR spectrum supports the other analysis methods in confirming the formation of hydrogel networks. In Figure 4, hydrogel displays the following corresponding signals [41,42]: the peak at 1.25 ppm corresponds to methyl (–CH_3_) proton (carbon no. 9,10,11,12); at 2.10 ppm for –CH- proton of (carbon no. 3, 5); at 4.70 ppm for –CH_2_– (carbon 4, 8). The proton of –NH and –COOH show strong peaks at 7.25 ppm and 8.10 ppm. Thus, the ^1^H NMR supports the FTIR in confirming the polymerization between DMAA and AMPSA.

### 2.5. X-ray Diffraction Analysis

**Figure 5:**
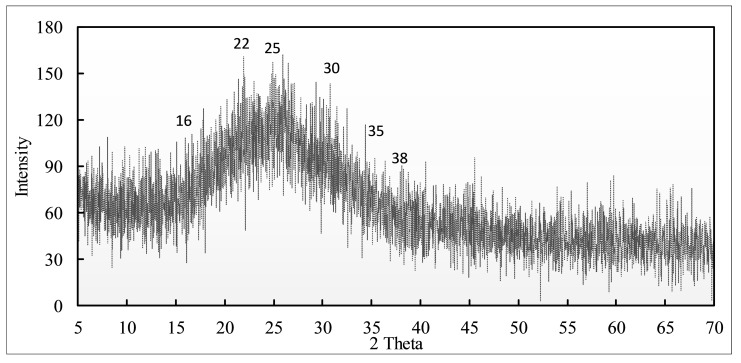
XRD spectrum of DMAA–AMPSA hydrogel.

The crystalline/amorphous nature of the gel was analyzed by X-ray diffraction run with a scene rate of 2 °C/minute with 2Θ ranges of 5 °C to 70 °C. Figure 5 displays the diffraction peaks for the gel prepared at 10 kGy radiation dose where DMAA–AMPSA does not show any sharp peaks but a broad noisy peak at 25 °C for the polymer. The other peaks from 16° to 38 °C (2Θ) may indicate the semi-crystallinity in the structure of the gel [43,44].

### 2.6. Thermal Analysis

**Figure 6:**
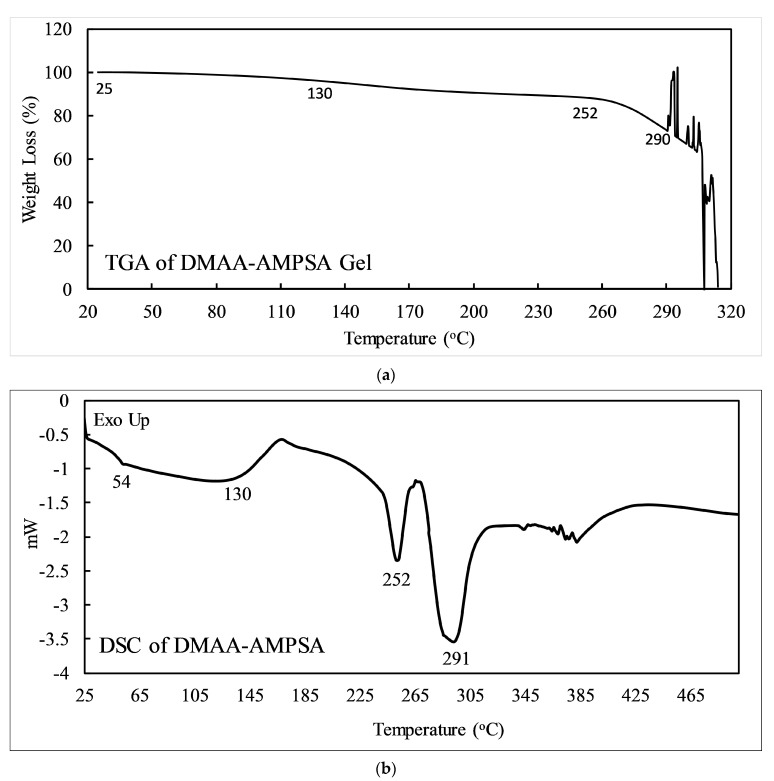
(**a**) TGA and (**b**) DSC thermograms of DMAA-AMPSA gel prepared by 10 kGy radiation dose.

Thermoanalytical technique differential scanning calorimetry (DSC) and thermo-gravimetric analysis (TGA) were carried out for the DMAA–AMPSA hydrogel prepared by applying a 10 kGy radiation dose shown in Figure 6. As a function of temperature, the DSC reflects the difference in heat flow between the sample and inert reference, where the TGA depicts the changes in sample weight. In Figure 6a—TGA graph—there are four stages of temperature changes. First, from 25 °C to 130 °C for dehydration; the second one is from 130 °C to 252 °C for changing to a glassy state; the third part is from 252 °C to 290 °C for melting, and the final stage is above 290 °C temperature; random changes in the graph indicate the degradation into other compounds which are explained in DSC. Elizabeth Elgueta et al. found similar TGA analysis results regarding the degradation of AMPSA-based gels [45]. In Figure 6b, the DSC thermogram depicts the initial decline of the graph, indicating the dehydration from the gel at around 54 °C, and the glass transition temperature (T_g_) is at 130 °C, which resembles the TGA graph demonstrating weight loss of up to 5% at the same temperatures. The melting temperature (T_m_) of the gel that appeared in the DSC graph is 252 °C, corresponding to the almost unchanged weight in the graph of the TGA. The last peak of DSC is at 291 °C; this temperature can be attributed to the evaporation of water due to the degradation of gel into new products/volatile compounds. The TGA graph clearly supports the situation at 290 °C by exhibiting drastic weight loss changes [46]. Above 291 °C, there is no smooth peak which indicates the degradation of the gel into smaller molecules. From both types of thermal analysis, it can be concluded that the prepared hydrogels are stable enough to use at room temperature.

### 2.7. Surface Analysis by SEM-EDS

**Figure 7:**
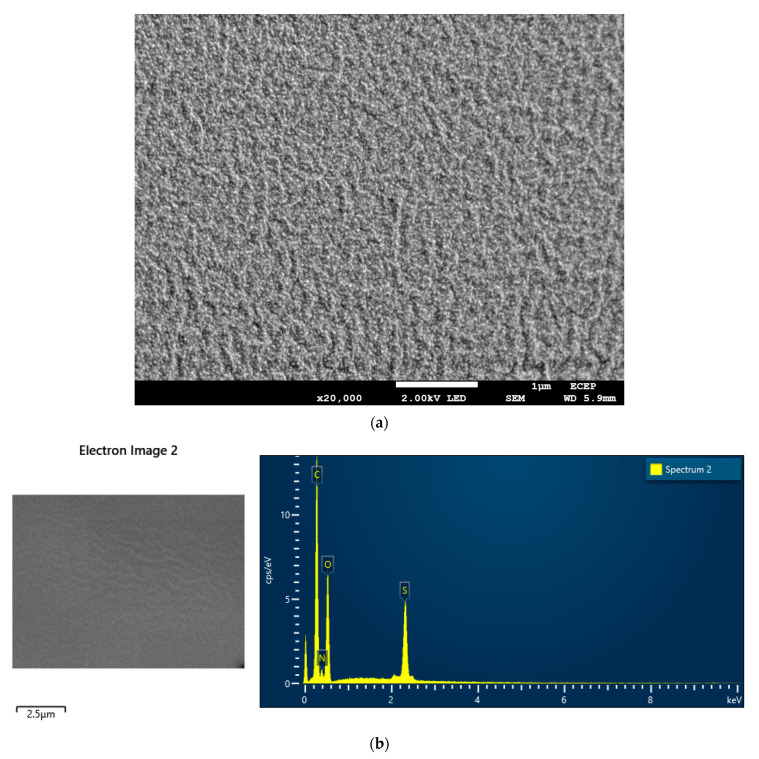
(**a**) SEM and (**b**) EDS of DMAA–AMPSA hydrogel.

SEM-EDS is a sophisticated qualitative analysis technique where the secondary and scattered electrons give rise image of the surface, and the X-ray detects elements present on that surface [42]. To investigate the surface nature and constitutional elements of the hydrogel, SEM-EDS was performed, as shown in Figure 7. Figure 7a exhibits the entangled surface morphology of the dry gel. There are pores in the structure where it can uptake solvent (water) as well as be capable of adsorbing other materials such as metal ions, dyes, and drugs. Figure 7b is clearly representing the elements carbon (C), nitrogen (N), oxygen (O), and sulfur (S) (Table 2), which consist of the polymeric gel. Therefore, the functional groups containing the above-mentioned elements, as well as carboxylic acid, amide, and sulfonic acid, are present in the polymer, which also supports FTIR and NMR analysis [47].

## 3. Conclusions

In this research work, DMAA–AMPSA hydrogels were prepared by applying different doses of gamma radiation ranging from 2 kGy to 30 kGy from the Co-60 source. The equilibrium swelling increases with increasing applied radiation dose up to 10 kGy, followed by decreasing due to the degradation of the monomers. The maximum swelling was found to be 26,324.9% of the dried weight of the gel at neutral pH and room temperature. The copolymerization of the monomers was confirmed by FTIR and NMR spectroscopy, which revealed the functional groups and the environment of protons. Thermal stability was analyzed via TGA and DSC thermograms, and it was found that the hydrogels were thermally stable up to 290 °C temperature. X-Ray diffraction peaks represented the semi-crystallinity/amorphous nature of the gel. The surface nature and the elements of the gel were confirmed by SEM-EDS, which indicated that the hydrogels possess porous entangled networks, and the elements carbon, oxygen, nitrogen, and sulfur resemble the elements of monomers. Therefore, it can be concluded that DMAA–AMPSA superabsorbent hydrogels are formed upon gamma irradiation in an aqueous solution and can be used in different fields of study. 

## 4. Experimental

### 4.1. Materials and Reagents

The 2,3-Dimethylacrylic acid and 2-Acrylamido-2-methyl-1-propane sulfonic acid were purchased from Sigma-Aldrich, Darmstadt, Germany. The ultra-pure water was used to conduct the experiments, and the temperature was kept at 198 K. The pH of the swelling media was accommodated by using nitric acid and ammonium hydroxide. 

### 4.2. Apparatus and Instruments

Basic laboratory apparatus (pH meter, oven, 3-neck round bottom flask, beaker, etc.) was used for conducting the experiments. The hydrogels were prepared by applying gamma irradiation from a Co-60 source, Center for Acceleration and Beam Applied Science of Kyushu University, Fukuoaka, Japan. Functional groups of the hydrogel were analyzed with Fourier transform infrared (FTIR) spectrophotometer (Thermo Scientific Nicolet iS50R FT-IR, Seoul, South Korea). Proton NMR of the sample was carried out by using JMTC-500/54/JJ, Tokyo, Japan. The crystallinity/Amorphous nature of the hydrogel was justified with X-Ray diffraction (XRD), Rigaku Smart Lab, Tokyo, Japan (Lamda = 1.54059 Angstrom). Thermal analysis was performed by using differential scanning calorimetry (DSC823e), with a scan rate of 5 °C/minute over the temperature range of 25 °C to 500 °C and N_2_ gas purging. Thermogravimetric analysis (TGA) (TGA 8000, PerkinElmer, Waltham, MA, USA) was carried out under a continuous N_2_ gas flow and heating rate of 5 °C/min over a temperature range from 25 to 300 °C. The surface nature and the constitutional elements of the hydrogel were investigated with platinum coating using scanning electron microscopy (SEM) (JEOL, JSM-7900F, Tokyo, Japan) equipped with an energy dispersive spectrometer (EDS). All of the characterization experiments were repeated several times to get precise results.

### 4.3. Preparation and Extraction of DMAA–AMPSA Hydrogels

At room temperature and atmospheric pressure, an aqueous blend solution of DMAA and AMPSA was prepared in a round bottom flask while stirring at 500 rpm. The solution was taken in a glass tube, followed by purging N_2_-gas to remove oxygen and air. Then, the sample tubes were subject to gamma irradiation of different doses (Table 3) from the Co-60 source for 24 h. The gamma source is the point source where the dose rate depends on irradiation time and the distance between the sample and the gamma source. Being completed irradiation, the hydrogels were taken out, cut into small parts, and dried at 50 °C temperature in the oven. During the preparation of hydrogels, a few monomers, water-soluble homopolymers, and degraded compounds may remain in the reaction system. That is why the specimens are needed to extract properly in ultra-pure water. For extraction, the dried samples were soaked in ultra-pure water in a beaker at 40 °C temperature for 24 h. Then, the samples were taken out and dried at 40 °C temperature in the oven until the constant weight was reached. At the time of extraction, the unreacted monomers and unwanted substances depart the hydrogel network.

### 4.4. Measurement of Super-Absorbency at Equilibrium Swelling

Equilibrium swelling of hydrogels was measured by soaking the dried samples in neutral pH media (pH = 6.5~7.5) at room temperature. After 24 h, the samples were removed, blotted with paper, and their weight was measured. To confirm the constant swelling, hydrogel’s swelling was continued up to 26 h, and weight was measured again every hour. Then, the equilibrium swelling was calculated from the dried and swelled weight by using the following equation:

(2) $$\mathrm{Water}\;\mathrm{absorption}\;\left[\%\right]=\frac{W_{t}-W_{1}}{W_{1}}\times 100\%$$

where *W*_1_ and *W_t_* are the dried-gel weight and the gel weight after swelling in the solution, respectively. The swelling experiment was performed three times.

## Figures and Tables

**Table 1:** Equilibrium swelling of a few APMSA hydrogels.

Hydrogel Name	Application	Maximum Swelling	Reference
Acrylamide/2-acrylamido-2-methyl-1-propanesulfonic acid/bentonite	Dye sorption	2282–12,603%	[26]
Salecan/poly(AMPS-co-HMAA)semi-IPN	Cell adhesion	5370%	[33]
AAm/AMPS and Semi-IPNs with PEG.	Water sorption and dye uptake	4850–22,560%	[34]
Terpolymer Hydrogels Based on AMPSA	-	2035 ± 255%	[35]
Poly (acrylic acid-co-2-acrylamido-2-methylpropane sulfonic acid)	Protein delivery	4715.2%	[36]
Superabsorbent composite hydrogels based on AMPS and acrylamide	-	7000%	[37]
Biodegradable Superabsorbent Hydrogels	-	9160%	[38]

**Table 2:** Composition of significant elements of hydrogel.

Element	Weight %	Weight % Sigma
Carbon (C)	53.42	0.58
Nitrogen (N)	11.19	0.79
Oxygen (O)	30.83	0.43
Sulfur	4.56	0.08
Total:	100.0	-

**Table 3:** Radiation dose and respective gel products for DMAA (10%) + AMPSA (10%).

Radiation dose (kGy)	2	5	10	20	30
**Dose rate (Gy/h)**	85	216	435	818	1302
**Distance from the source (cm)**	46	27	17	11	7
**Irradiation time (h)**	24	24	24	24	24
**Gel product**	Solid gel	Solid gel	Solid gel	Solid gel	Solid gel

## Data Availability

BHUYAN: MD (2023), “2,3-Dimethylacrylic acid- (2-Acrylamido-2-methyl-1-propanesulfonic acid) superabsorbent hydrogel”, Mendeley Data, V1, doi: 10.17632/6f2cgkv86x.1.
